# “More than just a walk in the park”: A multi-stakeholder qualitative exploration of community-based walking sport programmes for middle-aged and older adults

**DOI:** 10.1080/2159676X.2023.2197450

**Published:** 2023-04-02

**Authors:** Hamsini Sivaramakrishnan, Cassandra Phoenix, Eleanor Quested, Cecilie Thogersen-Ntoumani, Daniel F. Gucciardi, Boris Cheval, Nikos Ntoumanis

**Affiliations:** a Curtin School of Population Health, Curtin University, Perth, Australia; b Physical Activity and Well-Being Research Group, enAble Institute, Curtin University, Perth, Australia; c Department of Sport and Exercise Sciences, Durham University, Durham, UK; d Department of Sports Science and Clinical Biomechanics, University of Southern Denmark, Odense, Denmark; e Curtin School of Allied Health, Curtin University, Perth, Australia; f Swiss Center for Affective Sciences, University of Geneva, Geneva, Switzerland; g Laboratory for the Study of Emotion Elicitation and Expression, Department of Psychology, University of Geneva, Geneva, Switzerland; h School of Health and Welfare, Halmstad University, Halmstad, Sweden

**Keywords:** Healthy ageing, physical activity, team sport, walking football, ageism

## Abstract

In spite of the large-scale growth of walking sport (WS) programmes globally, limited research has explored the experiences of the key stakeholders involved in such programmes (i.e. decision-makers, facilitators, and players). We aimed to explore stakeholder experiences of community-based WS programmes to better understand the appeal of such sport options for middle-aged and older adults, and propose tentative recommendations for the feasibility and sustainability of these types of programmes. We conducted semi-structured interviews with 21 stakeholders who were involved with WS programmes in Australia as decision-makers, facilitators, and/or players. Data were analysed with reflexive thematic analysis. Four key themes pertaining to the WS experience were identified – ‘a renewed lease of life’, ‘navigating ageing stereotypes’, ‘tension between organisational demands and players’ needs’, and ‘WS facilitators as catalysts of success’. Specifically, we found that WS participation enabled a positive ageing discourse for middle-aged and older adults. WS players had to negotiate stereotypes that, at times, were perceived as participation barriers. We also noted some tensions between the demands of sport organisations and the needs of middle-aged and older adults regarding sport participation. Finally, we also noted the importance of the facilitators’ role in increasing accessibility of, and long-term participation in, such programmes. We suggest that to offer feasible and sustainable community-based WS programmes across Australia, incompatibilities across various stakeholders’ perspectives need to be addressed.

## Introduction

Growing numbers of older adults and increasing levels of inactivity with age have expanded the research interest in experiences of sport and physical activity in older age. Reviews of this work have indicated factors relevant to participation to be broadly grouped as those related to physical ability (e.g. pain), personal benefits (e.g. health, well-being), motivation and beliefs (e.g. self-efficacy, religious beliefs), competing priorities, social influences (e.g. interaction with peers, or health professionals), and access difficulties (e.g. affordability) (see Franco et al. 2015; Ige-Elegbede et al. 2019). Group-based activities in particular, have been shown to provide safe and inclusive spaces which promote feelings of social connection and belonging (e.g. Andersen, Ottesen, and Thing 2019; Hwang et al. 2019; Meredith, Witcher, and Wagstaff 2022), which is significant given the high levels of social isolation reported among older populations (Centre for Ageing Better 2019), and opportunities to socialise continually cited as a reason for participating in exercise groups/classes (Hwang et al. 2019).

In addition to focusing on the factors relevant to participation (often discussed using the language of ‘barriers and facilitators’), qualitative research has also drawn attention to the relational, embodied, gendered, and socially shaped dimensions of ageing and physical activity (e.g. see Franke et al. 2020; Griffin 2017; Oghene et al. 2015; Phoenix and Orr 2017). Such research has expanded knowledge into how these experiences are influenced by the various relationships occurring within these contexts. For example, this work has shown how health and exercise professionals leading group-based activities hold high levels of social power through their ability to impact the composition, atmosphere, and subsequent enjoyment of a group activity (Evans and Crust 2015; Morris, Guell, and Pollard 2019). It has also demonstrated the ways in which older adults’ experiences of physical activity are continually framed by broader social forces (e.g. ageism, sexism, ableism) that shape our expectations of what the ageing body can and cannot, or should and should not do. To that end, sport and physical activity has been found to act as an important vehicle through which older adults can *resist* but also *reinforce* ageist stereotypes that foreground the ageing process as a period of physical decline, deterioration, and social withdrawal (Horton et al. 2019; Phoenix and Smith 2011; Tulle 2008).

One type of group-based physical activity that have undergone rapid growth in recent years are walking sports (WS). These are adapted sports modified to reduce the cardiovascular demands on, and risk of injury to, participants (Reddy et al. 2017). Focusing instead on positioning, control, and motor skills, they provide an environment for social interaction (McEwan et al. 2019) and are well suited to those who wish to utilise their skills and remain physically active, without facing the vigorous demands that might accompany traditional sports played at their standard speed (Cholerton et al. 2019). WS programmes are prevalent worldwide, with noted presence in the United Kingdom, Europe, South Africa, Asia, North America, and Australia^1^ (Corepal et al. 2020). Walking soccer is the most prevalent WS with over 40,000 regular participants across 1000 clubs in England alone (Corepal et al. 2020). Findings from the relatively modest amount of research on WS accumulated to date, has shown that participation is often influenced by past sporting experience, perceptions of physical activity, and self-efficacy, with a supportive programme culture, and social and health-related outcomes being important for maintaining long-term engagement (Cholerton et al. 2021). It has also indicated that participants may have initial apprehensions regarding their capability to participate, as well as scepticism surrounding the appeal of the activity due to its slower pace (Reddy et al. 2017). Furthermore, UK-based pilot studies of walking football and netball have demonstrated the feasibility and acceptability of these activities among adults aged 50+ years (McEwan et al. 2019; Reddy et al. 2017), with an array of social-ecological factors spanning individual (e.g. health), interpersonal (e.g. social support), environmental (e.g. facilities), policy (e.g. funding), and global (e.g. marketing) levels being key factors for success (Kinnafick et al. 2021).

Importantly, existing WS research has typically been restricted to the perspectives of the players. The purpose of this paper is to further extend insights gained from this emerging body of research into the phenomenon of WS by incorporating the experiences of older players alongside those who serve as decision-makers and programme facilitators (in some instances in addition to playing the sport themselves). Being attentive to these often-neglected multi-stakeholder perspectives is of value because such qualitative work can enable a more holistic understanding of WS participation by combining player experiences with contextual insights into social-ecological facets of acceptability and feasibility relating to programme implementation and delivery. In other words, the relational and embodied aspects of WS participation can be considered alongside the practical and logistical issues involved with programme delivery. The findings of this paper are intended to help facilitate the future implementation of acceptable, feasible, and sustainable community-based WS programmes in a manner that encourages and enables long-term participation of middle- and older-aged adults.

## Methodology and methods

### Research paradigm

The present study forms part of a larger programme of research which aims to understand the potential of sport for promoting physical activity and mental well-being in middle-aged and older adults. This research includes a systematic review and meta-analysis of psychosocial outcomes of sport participation (Sivaramakrishnan et al. 2021) and a mixed-method study investigating the predictors of non-participant’s intentions to participate in WS, both of which were positioned in a post-positivist paradigm. That noted, as this ongoing programme of research has developed, there have been adjustments to the composition of the research team (the most recent of which including the involvement of an additional qualitative researcher). As a result, we have engaged in ongoing dialogue regarding the major issues confronting all research paradigms; we felt that the perspectives underpinning this current study were more closely aligned with those of constructivism. That is, we adopt a relativist ontology and subjectivist epistemology.

In their 5^th^ Handbook of Qualitative Research, Denzin and Lincoln (2017) note how over the past decade, the boundaries between the major paradigms and perspectives have begun to blur. Yet they also warn that ‘perceptions of differences between perspectives have hardened’, which can threaten to narrow the range and effectiveness of qualitative research practices (p. 98). We are aware that some of the positions on practical issues adopted by post-positivism versus constructivism differ, to the point of being incompatible. As examples, post-positivist inquiry typically aims to provide explanation of phenomena using prediction and control, whereas constructivism seeks to achieve this through understanding individual or collective reconstructions. Moreover, whereas post-positivist research draws on conventional benchmarks of ‘rigour’ such as internal and external validity, reliability, and researcher objectivity, constructivist inquiry favours trustworthiness and authenticity (Smith and McGannon 2018).

We do not see this shift into a constructivist paradigm for the most recent study as an absent-minded switching between seemingly incompatible assumptions regarding how we *do* research. Indeed, it is fair to acknowledge that some of the authors feel more at home here than others. Some will remain working within constructivism from herein – perhaps progressing further over their career into ever greater participatory, action orientated methodologies. Others might return firmly to the positivist/post-positivist beliefs that better align with their world view and continued pursuit of experimental, hypothesis driven, chiefly quantitative research in this subject area. However, in coming together as a group of disparate researchers, with varied expertise in methodologies and methods we settled on this qualitative exploration being rooted in constructivism. This ‘settling’ was achieved through the practice of what Mouffe (2000) refers to as agonistic pluralism, where the purpose is to acknowledge difference and not necessarily to reach consensus.

In line with the philosophical positioning and broader aim to understand and interpret the meaning of WS through the joint construction of meaning between multiple stakeholders, semi-structured interviews were the chosen method for data collection. Drawing on Sparkes and Smith (2013), these were well suited because they enabled participants to share the meanings they attached to their WS experiences, including their feelings and ideas about its acceptability, feasibility and sustainability. Further, the flexibility afforded by semi-structured interviews permitted participants to steer the conversation to share what is personally meaningful to them, rather than having a set of prescribed questions (and researchers’ underlying assumptions) imposed on participants’ narratives. This flexibility allowed the interviewer to ask unplanned questions iteratively based on participants’ responses, and for novel, and, at times, unanticipated insights to be gained.

### Participant recruitment

We obtained institutional ethical approval from Curtin University Human Research Ethics Committee (HRE2020–0742) and conducted our research in line with the appropriate ethical guidelines. These included, for example, voluntary participation, compensation for participants’ time, and ensuring participant confidentiality. Purposive criterion-based sampling and snowball sampling were used to identify participants (Sparkes & Smith, 2013,2022). Our inclusion criteria stated that participants must be: (a) decision-makers involved in WS at an organisational level and responsible for the implementation of these programmes, (b) facilitators responsible for carrying out day-to-day tasks pertaining to the delivery of community-based WS programmes, and/or (c) middle-aged or older adults who had any experience playing WS (i.e. players). We contacted decision-makers at state-level sport organisations across Australia that offered WS via email, provided an information sheet explaining the purpose of the study, and invited them for interview. We liaised with consenting decision-makers to identify facilitators and WS players who were willing to participate in an interview. To understand experiences of WS from multiple perspectives, we actively sought representation from a range of WS (e.g. soccer, netball, hockey) and roles (decision-maker, facilitator, player) in our sample. Similar to Braun and Clarke (2019b), we rejected the neo-positivist positioning of the concept of saturation. Instead, we focused on information power, or the richness of data obtained, rather than the number of individuals interviewed (Malterud, Siersma, and Guassora 2016).

In total, 21 participants (10 males and 11 females) across five states and territories in Australia (i.e. Australian Capital Territory, New South Wales, Queensland, South Australia, Western Australia) were recruited. Although we set out to recruit from distinct roles as noted above, we found that individuals commonly fulfilled multiple roles. For example, some participants regularly played WS, occasionally facilitated sessions, and/or contributed in an administrative capacity. WS players were aged between 53 and 72 years. To avoid potential identification of participants, given the limited number of organisations currently offering WS in Australia, we provide group-level demographic information (see Table 1).

**Table 1.** Demographic information for interviewees.

Variables	Values
*Continuous variables*	*Mean (Standard Deviation)*
Age (for participants, in years)	62.25 (6.30)
Duration of walking sport participation (for participants, in years)	1.21 (0.85)
*Categorical variables*	*N (%)*
Gender	
Male	10 (47.6%)
Female	11 (52.4%)
Role*	
Decision-maker	9
Facilitator	10
Player	12
Sport	
Soccer	14 (66.7%)
Netball	4 (19.0%)
Hockey	3 (14.3%)
Prior experience playing sport (for participants)	
Yes	8 (66.7%)
No	4 (33.3%)
Prior experience playing sport (for decision-makers and facilitators)	
Yes	14 (100%)
No	0 (0%)

*Interviewees, at times, fulfilled more than one role. Therefore, the *N* presented refers to the number of interviewees who fulfilled a specific role, and hence, it was not possible to report percentages for this variable.

### Data collection

Initially, we developed three interview guides – one for each category of participants interviewed. Each contained distinct questions that a specific stakeholder category might have been uniquely positioned to answer. However, in response to participants often fulfilling multiple roles, the interview guides were revised to provide a pool of questions that were drawn upon according to the role(s) of the person being interviewed. This allowed us to get a sense of the participant’s personal experience, alongside (where relevant) organisational demands, and other implementation considerations. As the present study was exploratory, questions were open-ended, enabling participants to guide the narrative. HS piloted each topic guide prior to commencing data collection to ensure that questions elicited rich description. Key topic areas and exemplar questions for each interview guide are presented in Table 2.

**Table 2.** Interview topic guides – topic areas and exemplar questions.

Interviewee	Topic Area	Example Question
Decision-makers	Popularity of walking sports	Can you tell me a little bit about the walking sport programmes that your club/organisation offers?
	Organisational demands and expectations	What kinds of resources does the organisation need to arrange for and contribute towards these walking sport programmes?
	Practical considerations for implementation	From your perspective, what is needed to ensure these programmes are viable and sustainable in the future?
Facilitators	Setting up and delivering walking sport programmes	How would you describe your relationship with participants of the programme?
	Responsibilities of the facilitator	As a coach, can you tell me a little more about what your responsibilities are for delivering walking sport programmes?
Participants	Sport history	Can you describe your past experience with physical activity?
	Motives for participation	What attracted you to start participating in walking sport?
	Experience and intentions to participate in the future	How did you feel after participating in walking sport for the first time?

Using the interview guide, HS conducted interviews using either telephone or Microsoft Teams video call facility, in line with Government COVID-19 protocols at the time of interviewing. At the end of each conversation, the participant was asked if there was any other relevant information to consider that had not been discussed, and the interview concluded when the participant was satisfied that all pertinent information was provided (Krueger and Casey 2015). Interviews lasted approximately one hour (ranging from half to one-and-a-half hours). Following each interview, HS recorded hand-written notes of additional details such as personal reflections and participants’ non-verbal cues. These notes provided insight that helped navigate subsequent interviews. For example, HS regularly discussed her personal reflections with EQ, who was then able to recommend specific matters of interest that may be worth probing further in subsequent interviews.

### Data analysis

We analysed the data iteratively and inductively using reflexive thematic analysis (RTA) as outlined initially by Braun and Clarke (2006), and subsequently refined in the years that have followed to emphasise the researchers’ role in knowledge production (see Braun and Clarke 2019a, 2021). The theoretical flexibility afforded by RTA offered the possibility of inductively analysing data – we explored semantic and latent meanings through illustrative and analytical treatment of data. This theoretical flexibility was especially important when we were interpreting data that was obtained from individuals who held multiple roles as it was particularly challenging to discern the perspectives of specific ‘types’ of stakeholders. Therefore, RTA enabled the positioning of personal experiences within the prevailing social context (Braun and Clarke 2021). Equally, the accessibility of RTA was also important as it enabled the research team, encompassing researchers with a wide range of expertise, some of whom had no prior experience of qualitative research, to contribute to the analysis (Braun and Clarke 2022). In keeping with our interpretivist positioning, RTA allowed us to interpret context-specific meaning and expand our understanding of WS, specifically in terms of addressing our research aims (Braun and Clarke 2021).

To increase familiarisation with the data, HS transcribed all interviews verbatim, re-read the transcripts, and maintained reflection notes to record personal interpretations of the data. Given the limited research on multi-stakeholder experiences of WS, we adopted an inductive approach during the coding process (Braun and Clarke 2019a). To enhance reflexivity through the analysis, HS provided CP and EQ with transcripts of the conducted interviews. We subsequently discussed our individual interpretations of the data – this process helped us improve the richness of our overall interpretation. HS drew out semantic and latent open codes iteratively. Latent codes were drawn out upon reviewing interview transcripts and interview notes several times, discussing interpretations with co-authors, complemented by an engagement with prior literature that had relevance to our interpretations of the data.

Codes were revisited to identify patterns and to begin to interpret meaning across the data. This process enabled the development of initial themes. Often transcripts were re-read and additional codes were assigned following initial theme development. For example, we had developed some initial codes labelled as ‘being active’, ‘self-efficacy for sports’, and ‘sport identity’. Upon revisiting the codes, we identified that these ideas shared a central organising concept in that these ideas were discussed by participants in relation to age-related participation benefits. As such, these codes were clustered together and labelled as ‘age-related benefits’. Similarly, ‘catering for diversity in needs’ was originally a separate theme. However, upon discussion, we agreed that this fitted as a subtheme within the overarching ‘tension between organisational demands and players’ needs’ theme; we noted that the codes pertaining to catering for diversity reflected divergent perspectives, one important aspect of the noted tension between stakeholders competing needs and demands. Through this process, ‘competitiveness’ was separated from ‘being active’ as these codes seemed to be capture two distinct meanings (i.e. intrinsic and extrinsic participation motives).

During this analytic process, we drew thematic maps, which were continually reviewed and revised throughout. We then reflected upon the essence of each central organising concept to drive the definitions and names of themes to build an informative narrative that reflected the data. For instance, we renamed a theme originally called ‘practical considerations’ to ‘WS facilitators as catalysts of success’ to provide a more informative description of the subthemes that reinforced the key role of programme facilitators. Finally, to facilitate the writing-up phase, we identified appropriate participant quotes which represented different aspects of each theme, and contextualised our findings in relation to relevant literature.

## Methodological Rigour

To support rigour, HS maintained a reflexive journal throughout the analysis process. For instance, while several authors have participated in community-based sports, none of the authors had any involvement or experience with WS. Further, HS, who was responsible for drawing out codes, was a young adult. Journaling helped us better understand and challenge our personal assumptions relating to WS and sport participation in ageing adults, and enabled us to draw connections between our assumptions and interpretations of the data (Braun and Clarke 2019a). Following Levitt et al. (2021), our intention in considering multiple perspectives (i.e. decision-makers, facilitators, players) was not a positivistic attempt to find ‘truth’ through triangulation, but to support fidelity by enhancing our understanding of the diversity in experiences. Throughout the analysis process, HS sustained regular discussions with CP and EQ who were experienced in qualitative research and served as ‘critical friends’. This was not to ensure accuracy of codes, but rather to encourage reflexivity in our analysis (Smith and McGannon 2018). Thick descriptions were used to contextualise the findings within the participants’ unique positioning (Levitt et al. 2021).

## Results and discussion

Four key themes were identified to provide insight into individuals’ experiences regarding the acceptability, feasibility, and sustainability of community-based WS programmes. These were ‘a renewed lease of life’, ‘navigating ageing stereotypes’, ‘tension between organisational demands and players’ needs’, and ‘WS facilitators as catalysts of success’. The first two themes broadly relate to the acceptability of WS in the context of ageing, whereas the second two themes relate to the feasibility and sustainability of community-based WS programmes. Together, these themes provided unique knowledge of WS experiences that could be used to inform recommendations with a view to optimising the implementation of future WS programmes.

### A renewed lease of life

Reflecting on their experiences with WS, in the context of ageing, some WS players repeatedly referred to a sense of youthfulness attributed to their involvement in ‘active’ sports. Luke (pseudonyms used throughout), a 62-year-old walking soccer player and decision-maker remarked:

*The next game that would be best suited for me would be lawn bowls … I’m much too young for that. I’m definitely much too active … there’s not been an intermediary game from playing football. This is good … which is why we all do it.*

Such appraisals of youthfulness may facilitate WS participation, especially for individuals who identify as being ‘physically active’. The idea of WS being an ‘intermediary game’ highlights that the moderate pace could cater to a wide range of fitness levels and abilities. Luke also appeared to adopt the view of sport being ‘good’, suggesting that WS participation is associated with socially desirable personal attributes. In western society, physical activity is a socially desirable behaviour, with a significant moral worth placed upon sport participation (Gard et al. 2017). The moral worth of sport seemed to motivate Luke to remain active through WS participation – he perceived himself to be ‘ageing successfully’.

Another participant, Alfred, who played WS and served facilitator and decision-maker roles, expressed feeling a sense of confidence as a competent player during his transition from Masters’ soccer to walking soccer at age 65:

*I’ve realised that playing within my own age bracket of over 60s, all of a sudden, I was a good player again, because I wasn’t having to compete against really young guys … playing within your own capabilities and within your own age bracket, all of a sudden, if you’re a decent player, you’re a decent player again, and that was good for me personally.*

A shift in playing environment appeared to permit favourable comparison with peers, or perceiving oneself to be more youthful than others of a similar age. Stephan et al. (2013) suggested that favourable self-perceptions are associated with rejecting negative age-related stereotypes, higher self-efficacy, intentions towards physical activity, and better physical functioning. For ageing adults who had previously played sport for a significant period of time, WS seemed to provide an opportunity to continue participating in sports in a less physically demanding manner. Such opportunities may enable players’ experience of self-efficacy related to their athletic skill, which has been noted as a key facilitator for sustaining sport participation (Whitehead et al. 2019). Furthermore, sport participation may enable ageing adults to develop a positive ageing discourse (Jenkin et al. 2017). As individuals got older and felt limited in their ability to participate in enjoyable physical activities, WS seemed to fill a potential void, highlighting the potential identity continuity provided by such sport opportunities (Dionigi, Horton, and Baker 2013; Walsh et al. 2018). In Alfred’s case, the feeling of being a ‘decent player’ seemed central to retaining a sport identity. McEwan et al. (2019) indicated that a ‘sport identity’ sustained through WS participation may provide a purpose or ‘a new lease of life’. Such sense of purpose has been identified as relating to positive health practices such as making improved food choices and physical activity (Walsh et al. 2018). Thus, WSs can accommodate change in a manner that, over time, traditional sports may be less able to and allow the ageing body to be responded to appropriately.

### Navigating ageing stereotypes

Responses from stakeholders often highlighted (subliminally) the presence of ageism within WS, which, at times, were viewed by players as a barrier to overcome. For example, while describing the potential benefits of WS for ageing adults, one decision-maker (Graham) referenced a commonly-held negative stereotype about ageing and health outcomes, by saying ‘we don’t want them being a burden on the medical system’. These (sometimes inadvertent) micro-aggressions were identity damaging and reflected the neo-liberal shift in language related to the ‘healthy ageing’ discourses predominant in sport and health promotion policies. Such discourses place the burden of an individual’s health on their own choices and actions (rather than social structures and inequalities) (Gard et al. 2017) – sentiments also reinforced by the Exercise is Medicine movement (Pullen and Malcolm 2018).

Also showing how decision-makers and facilitators can play a role in sustaining such age-related stereotypes, when discussing the scheduling of walking netball sessions, one facilitator, Lily, remarked:

*A lot of people of the demographic doing WS really probably have quite a bit of time … when you kind of look at their schedule compared to mine, it’s like, you probably could move lunch half an hour later.*

This view highlighted an underlying assumption that ageing adults lacked other competing responsibilities, when in fact, older adults can have busy lives – remaining in paid employment, keeping up with various engagements, fulfilling non-negotiable informal caregiver roles for elderly relatives and grandchildren, or simply pursuing other interests (Jenkin et al. 2016).

At times, WS players also showed that ageist stereotypes may have been incorporated into their sense of self. Patrick, a 72-year-old player, expressed:

*At my age, I don’t want to feel the pressure of winning. I don’t enjoy it if we don’t win and there’s a large amount of disappointment. At my age, I just want to participate.*

Patrick seemed to attribute his desire to participate without ‘the pressure of winning’ to his age, suggesting that others of a similar age may share his sentiment, and a desire to win may be more age-appropriate for younger players. Perceived societal expectations regarding participation in age-appropriate activities have been found to significantly influence older adults’ sport participation (Jenkin et al. 2016). Through continued experience over the life course, societal expectations regarding age-appropriate activities may frame self-perceptions of ageing, which are believed to shape how people give meaning to notions of health, including their participation in health practices such as physical activity (Emile et al. 2014).

Within this theme, we also noted players, particularly those who had played sport previously, highlighted the stigma surrounding the label ‘walking sport’. Several WS players recalled feeling an initial lack of interest towards trying out the WS available in their local community as a result of the label ‘walking’ sounding ‘unappealing’ or ‘geriatric’. The slower pace seemed associated with lower mobility and senility. As such, this was deemed to be one of the biggest barriers to attracting new players to the sport, especially those who did not consider themselves to fit this prevailing age-related stereotype (see Horton et al. 2018). Keith, a 65-year-old player who had previously played traditional soccer to a semi-professional level, explained:

*When I was first told about it, I turned my nose up, I’d never heard of it, I’d never seen it. I thought, “walking? What good is that?” And so, I was quite negative, really bad at first …*

However, an overwhelming majority of players who were able to move through this initial barrier, typically by going to watch a session or by accompanying a friend who was already a regular player, reported thoroughly enjoying the experience, and often became regular players themselves (as discussed in ‘A renewed lease of life’). Keith went on to add:

*… but then when I went and I tried it, I thought, yeah, it brings back some memories in there and you could try a few little things that you used to do years ago.*

This common pattern showed how players may have navigated age-related stereotypes pertaining to sport participation. To create coherence between his self-identity and pre-conceived negative stereotyping of WS and their typical players, Keith may have distanced himself from this notion, believing that he was an ‘exception to the rule’ (p. 36); that such stereotypes applied to others rather than himself (see Horton et al. 2018). In such instances, these self-serving views of ageing may deter potential players from initiating WS participation.

Stakeholders also noted that WS programmes tended to *retain* a large number of those who came to try a session, but the largest barrier remained *initiating* interest. One walking hockey facilitator, Mitchell, wondered whether changing the name might eliminate this barrier:

*They [potential players] believe they’re still eighteen and they can still run … Maybe having the term walking in there is negative. Maybe there’s something else that can be supplemented for it … to make it seem like it’s more than just a walk in the park.*

This view also seemed to echo players’ association of fast-paced sport with younger ages and emphasised the need for programme marketing to reflect the move away from discourses of ageing as a period of decline. That said, due to the widespread growth of WS as a global brand, stakeholders believed it was too late to change this label. Rather, Mitchell added that, instead, the focus should be on addressing the stereotype rather than changing the name, by improving the visibility of the sport ‘so people realise it’s actually a lot more fun, and a lot more challenging than it sounds’. Improving visibility may have positive implications for ageing adults’ perceptions and awareness of, as well as self-efficacy for, WS, while minimising anxiety and apprehension surrounding WS participation. These factors are known to encourage the initiation of WS participation (Cholerton et al. 2019). Reflection is encouraged within the organisational structures regarding the extent to which players and staff might be (inadvertently) complicit in the negative stereotyping of ageing adults, as these may be enduring factors limiting participation.

### Tension between organisational demands and players’ needs

When comparing stakeholders’ experiences, some points of divergence were noted, particularly in relation to the visibility of WS, diversity in players’ needs and motives, and competing interests of stakeholders and funders.

Firstly, interviews with decision-makers revealed how tensions were experienced relationally and seemingly accentuated discrepancies between stakeholders’ beliefs around aims and actions of the programme. For example, decision-makers in charge of implementing WS in local communities described the desire to promote physical activity via WS. This was shown in comments made by Will – a WS player, also involved with implementing and delivering a walking soccer program, who when describing the purpose of WS explained:

*[WS is] to give people who are otherwise socially isolated and inactive a bit of an outlet … a chance to kind of engage in a bit of social activity.*

In contrast, however, a different perspective was offered by Kate, another walking soccer facilitator and decision-maker, who noted that the ‘main aim [of WS] is retention … to give people an opportunity to remain in the game’, rather than to engage inactive individuals, or promote physical activity more broadly. This aspiration was reinforced by observations that a large proportion of publicity surrounding WS offerings was circulated among a database of ex-players, thereby limiting the visibility for, and inclusion of, those outside these networks. Incongruence between stakeholders’ perspectives have been observed in other physical activity contexts such as prescriptive General Practitioner exercise referral schemes (Buckley et al. 2019). The prescriptive nature of these schemes may limit their acceptability to service end-users; instead, co-production, or an ongoing reciprocal interaction between key stakeholders, would support congruence between programme planning, delivery, and reception (Buckley et al. 2019). For example, Jimmy expressed a typical perspective among players regarding the lack of visibility of programmes:

*I would’ve had no idea that this was available to me unless I’d heard through word of mouth. I didn’t see any signs, any promotion, any advertising … but I enjoy it, I have a bit of fun, and I’m happy to keep going now for as long as I can.*

However, decision-makers and facilitators seemed perplexed. Jake, a player and facilitator, remarked:

*I don’t know what else we can do. I think there’s even been something on one of the news programmes … I know I’ve seen it in the [newspaper] … all the media methods seem to have been used, but I don’t know how we encourage more people.*

These perspectives highlighted the stark contrast among stakeholders’ perspectives, with such a disconnect potentially having a negative impact on the longer-term implementation and uptake of such programmes. Although sport organisations were largely aware of the benefits of sport participation for ageing adults, facilitating such opportunities remained a low priority (Jenkin et al. 2021). The resulting lack of awareness was a major barrier influencing the uptake of physical activity interventions among the older age group (Crozier et al. 2020). These challenges related to visibility, particularly the lack of marketing of sport programmes directed towards insufficiently active individuals, emphasise the need to consult with experts bearing a specific understanding of the target group (Staley et al. 2019). Further, increased participatory research in the implementation phase would ensure understanding of individuals and communities relative to such community-based sport programmes (Kinnafick et al. 2021).

A second tension highlighted through the analysis was the need for WS programmes to cater for the diversity in players’ needs and motives. Troy, a decision-maker and facilitator, remarked:

*I think when you’re a 74-year-old, you just wanna have a kick or laugh, the result’s not that important, as long as you’re part of something … They’re not as results or technique driven as younger players.*

His comments reflected the general consensus among decision-makers that older adults had intrinsic motives for participation (e.g. enjoyment) and were motivated by a desire to participate, rather than a desire to compete or win. This assumption has been critiqued by numerous scholars working with older adults involved in sport/physical training who can remain fully committed to beating opponents and achieving personal bests as they age (see Phoenix and Smith 2011; Tulle 2008; Dionigi, Horton, and Baker 2013). There is an increasing need to recognise the heterogeneity of this demographic group. As they age, adults may hold a variety of participation motives; extrinsic motives driven by competition and testing physical limits, but equally, intrinsic motives for social connection and affiliation (Molanorouzi, Khoo, and Morris 2015; Stenner, Buckley, and Mosewich 2020). In the present study, differences in players’ preferences for competition was typically linked with their prior experience with sport. Those with several years of sport experience seemed to express a liking for competition, while novice players seemed to prefer non-competitive environments. Luke, a walking soccer player with several years of soccer experience, said:

*It is competitive … that’s why everybody wants to play, we all played [soccer] previously … You don’t play [soccer] not to be competitive.*

Meanwhile, Christine, a novice player, said:

*I want to be in an environment where the people around me don’t mind that [I’m not competitive], if they mind that, then I’m not comfortable playing sports with them.*

The diversity in motives was a noteworthy tension that some facilitators struggled to reconcile, but, equally, was seen as something warranting action. One facilitator and player, Mary, recalled how they successfully navigated this situation:

*We set up two pitches or two fields, one is for competitive, one is for people that don’t want to be competitive … If someone comes along to watch … it’s like a shop window … they’ve got a couple of options as to what they can do. [Previously] we’ve had one or the other only, and it’s probably put some people off.*

This view acknowledged the heterogeneity of ageing adults’ motives, and suggests one technique to successfully accommodate differing needs. It also highlights the consequences of failing to do so; individuals experiencing an incompatibility between their preferences and the programme offering tended to disengage. As such, the uptake of WS programmes relies on varied participation preferences being successfully accommodated (Kinnafick et al. 2021).

A third tension related to concerns expressed regarding competing interests of government bodies, other funding bodies, and sport organisations. In Australia, funding was awarded to a national sport organisation to implement WS targeting the 55+ age group. However, some facilitators disagreed with an age limit being imposed, highlighting numerous reasons why individuals may participate in WS. Troy explained:

*The grant funding is aimed at 55 + .So, we report on that number to the government and [national sport organisation], but for me it’s [sport] for anyone that can’t play a regular format, so you might be coming back from a knee reconstruction … You might be overweight and want to find a low impact way to get into sport again.*

This view highlighted the influence of centralisation, or the increased national direction, on the implementation of community-based sport programmes. A trickle-down effect of nationally established policy regarding sport participation influences the focus of sport organisations, which in turn drives local community sport development targets (Dionigi et al. 2021). As such, centralisation may limit the autonomy of local sport organisations to adequately address community-specific needs (Misener et al. 2022). Instead, fostering interorganisational relationships may be vital to decentralisation, and moving towards collaborative approaches serving the needs of various stakeholders (Misener et al. 2022).

Regarding the growth of WS, a sense of frustration with sport organisations was noted. Jake, a player and facilitator, remarked:

*I don’t think [sport organisation] had done as much as they could do, because they also run Masters [sport], and they could lose people there. Unfortunately, sometimes it’s people who think that they can see a dollar to be made rather than what they can do for the sport.*

This view emphasised the negative impact of commercialisation of sports and the conflict of interest that may arise from centralisation. A strategic focus on more lucrative target groups, such as youth and elite athletes poses a key organisational barrier to promoting older adults’ sport participation (Jenkin et al. 2016). Collaboration and engaged partnerships between sport organisations and other policy sectors may aid the sustainability of community-based physical activity programmes (Estabrooks et al. 2011).

### WS facilitators as catalysts of success

Participants discussed the importance of the facilitator’s role in driving the accessibility and successful delivery of WS. Largely these discussions focused on how a facilitator may be distinctly positioned to understand the unique conditions that exist within their specific WS program, and therefore able to make appropriately informed decisions to support players’ needs.

For instance, Amy, a facilitator highlighted a significant barrier in the reliance on technology, requiring players to register using a web-portal:

*I guess my boss’s perspective was they just need to register online, they just need to learn how to do it in this day and age. But then actually talking to them, some of them just don’t have the capacity to go online, remember passwords and such … that’s one of the main challenges.*

This view illustrated a disconnect between decision-makers and players, where facilitators may serve to bridge the gap. In this instance, the decision-maker suggested that ageing adults simply needed to keep up with the times, failing to acknowledge structural and personal limitations, and dismissing facilitators’ feedback. Yet, facilitators of that programme ensured that interested players were included regardless of whether they had registered online, illustrating an instance where facilitators may have been better placed to understand and accommodate players’ requirements.

Facilitators highlighted the importance of cultivating a need-supportive atmosphere. One facilitator, Lily, recalled an example of how she was able to use her professional knowledge as an exercise physiologist to support players:

*A lot of them [say], “no, no, I have arthritis, I can’t do that” and I’m like “It’s okay, you can. I know what that is. I work with that a lot. Trust me, I’m not gonna do anything that’s gonna hurt you”. And then once one of them starts talking about their injuries, the rest of them join in, and it’s like, “okay, we’re all a little bit broken. We can do this”. So, it’s kind of nice.*

This view exemplified the importance of listening, acknowledging feelings, and reassuring players, while adapting sessions to suit players’ skill-levels to ensure that WSs were accessible to members of the community regardless of their skill or ability. Feeling ‘broken’ was reframed from being perceived as an individual weakness, to a collective identity within the group, where disclosure was shared and players potentially felt supported in each other’s company. These behaviours seemed to cultivate a sense of competence and relatedness among players. Such competence need-supportive behaviours demonstrated in a group exercise context have been regarded as effective in creating positive motivational experiences for players and maintaining participation (Hancox et al. 2018). Need-supportive communication, involving empathy, patience, and flexibility, may allow facilitators to understand players’ perspectives and ensure these are given due consideration by other relevant stakeholders (Ntoumanis et al. 2018). In an investigation of a community-based netball programme, Walsh et al. (2018) also echoed the importance of the facilitator in fostering opportunities for players to experience competence and relatedness within the group. Such experiences of competence and relatedness may ultimately foster higher autonomous motives, and lower controlled motives, for the activity (Ntoumanis et al. 2021), which in turn is related with positive physical and mental health outcomes (Ng et al. 2012).

A decision-maker and facilitator, Troy echoed this sentiment of ‘fit’ between facilitators and players:

*We’ve had a [younger] person running it, that wasn’t that interested or was just doing it to tick a box, not because they cared, so the key factor in football is to have a champion, someone that’s [older], passionate about this demographic, that wants these programmes to succeed.*

This view suggested that facilitators’ age may be implicated in this ‘fit’ – players may be further encouraged to participate in WS if they personally identified with the facilitator. The importance of having a ‘champion’ was supported by Kritz et al. (2020) who highlighted the influential role of peer leaders in promoting physical activity among individuals of a similar age. Equally, this ‘fit’ may also have to do with ‘interest’, ‘care’, and ‘passion’ for the demographic group, which, ultimately, were credited for the success of such programmes. This perspective was supported by Kate, a walking soccer decision-maker and facilitator:

*That’s probably a little bit unique to my program, only because I am so involved in it … I think the main reason for me is how I saw what it could do for my dad, and I thought this is an amazing thing, and I want to be a part of it.*

This view emphasised the necessity of buy-in from someone in a facilitator role. In line with this perspective, facilitators would need to understand the target population, as well as the benefits of, and barriers to, participation for these individuals (Staley et al. 2019). In this instance, Kate’s understanding of the benefits of WS for the target group seemed to provide a greater purpose and motive to deliver such programmes. Taken together, facilitators may serve a pivotal role in the success of community-based WS programmes.

## Concluding remarks and future directions

One of the key strengths, given the exploratory nature of our research, is that our inductive approach to RTA has allowed us to highlight aspects of acceptability, feasibility, and sustainability of WS programmes that stakeholders consider to be important. Correspondingly, this research involved participants of diverse backgrounds, representing perspectives of various stakeholders (i.e. decision-makers, facilitators, and players) of community-based WS programmes across Australia. This enabled a multi-faceted understanding, highlighting the diversity and, at times, incompatibility of the perceptions of such programmes (e.g. participation motives). Our findings extend prior qualitative literature on physical activity and ageing, suggesting that: (1) ageism remains a challenge that stakeholders need to navigate, (2) stakeholders may, at times, hold incompatible perspectives, and (3) facilitators may serve an important role in bridging the gap between decision-makers’ and players’ perspectives.

However, we acknowledge that our findings need to be viewed in context. The stakeholder participants in the present study were all involved in WS at the time of interviewing, and therefore, perhaps had a vested interest in WS and ensuring its continuity. We were unable to identify and interview individuals who had dropped out of WS, or those who perceived WS to be unappealing, who may presumably have different experiences with (or views of) WS.

Facilitators play a vital role in driving programme success and may be regarded as gatekeepers of information pertaining to the WS programme for which they are responsible. Facilitators’ insight into players’ feedback and relationships with decision-makers may be useful to help reconcile the noted tension among the various stakeholders of WS. Decision-makers may wish to take on board facilitators’ feedback to iteratively improve the delivery of WS programmes. Some examples of this feedback may be in reducing the reliance on technology for programme registrations, expanding programmes to players of all ages, finding ways to bridge the gap in programme visibility, and even understanding the importance of skilled facilitators who buy-in to the outcomes that WSs can offer to the community.

Although we also emphasise that facilitators played a crucial role in programme success, there is a caveat – in practice, facilitators were typically unpaid volunteers and challenging to recruit. As such, inadequate support and compensation may place an undue and unrealistic burden on facilitators to deliver successful programmes. In the absence of sufficient funding to compensate facilitators for their time, engaging volunteers may be one potential solution. Yet, identifying facilitators/volunteers who are appropriately skilled and have the time and capacity to deliver such programmes may be a key challenge to ongoing delivery of non-traditional sport programmes for inactive adults (Staley et al. 2019). Future research may wish to consider how facilitators and volunteers may be effectively, feasibly, and sustainably engaged to deliver community-based physical activity programmes.

Although we highlight key stakeholders’ stereotypes as relevant to physical activity and sport participation in ageing adults, ageism is a global issue that infiltrates all aspects of society and goes beyond WS (World Health Organisation 2021). A complex interplay of factors, across personal to systemic levels, may be implicated in the persistence of these issues (Swift et al. 2017). For example, the incongruity between key stakeholders is a noteworthy challenge, that may limit the acceptability, feasibility, and sustainability of WS programmes in their current form. This incongruity may be, in part, because the implementation and delivery of WSs have relied on administrators within sport organisations who typically did not belong to the target population of WS, which, in turn, could further exacerbate the pervasiveness of ageing stereotypes held by key WS stakeholders. As these prevailing ageing stereotypes fail to acknowledge the varied ways of ageing, there is a need to dismantle the dominant narratives of decline. Institutions, including government and sport organisations, should reflect on policies and practices that may sustain ageist beliefs and/or systematically disadvantage ageing adults’ access to appropriate community-based physical activity opportunities. Such stereotypes of ageing (including internalised stereotypes) could be addressed through the introduction of counter-stereotypical messaging and imaging to normalise physical activity in ageing adults. While a systematic review of interventions that addressed age-related stereotypes indicated reductions in negative views-on-ageing and increases in older adults’ physical activity (Knight et al. 2022), future research may wish to consider whether such interventions may bear utility in diminishing stereotypes held by other key stakeholders. Equally qualitative work could be furthered in this regard, drawing attention to adults’ views and experiences of age-related stereotypes in relation to health and physical activity.

## Note

1. At present, in Australia, WSs have received funding from the Australian government, are broadly driven by state sporting organisations, and delivered at local sport clubs as community initiatives to engage individuals who may be unable to participate in their traditional sport offering (SportAus n.d..). Typically, these programmes are mixed-gender and provide flexibility through a Pay As You Go structure (Walking Netball n.d..).
